# Developing a prospective ethical governance framework for a discrete choice experiment involving children aged 6–12 years: the PREF-ANX Kids study

**DOI:** 10.1186/s12910-026-01565-6

**Published:** 2026-09-23

**Authors:** Inês Martins Esteves, Márcia Pestana-Santos, Filipa Sampaio, Margarida Reis Santos

**Affiliations:** 1 Department of Public Health and Caring Sciences, Uppsala Health Economics, Uppsala University, Uppsala, Sweden; 2 School of Medicine and Biomedical Sciences, University of Porto, Porto, Portugal; 3 Nursing School, RISE-Health, University of Porto, Porto, Portugal; 4 UICISA:E – Health Sciences Research Unit: Nursing, Coimbra, Portugal; 5 Nursing School, University of Coimbra, Coimbra, Portugal; 6 Nursing School, University of Porto, Porto, Portugal

**Keywords:** Assent, Children’s participation, Data protection, Discrete choice experiment, Paediatric research ethics, Paediatric preference research

## Abstract

**Background:**

Discrete choice experiments (DCEs) are quantitative preference-elicitation methods, developed largely within health economics, in which participants choose repeatedly between hypothetical scenarios composed of systematically varied attributes. Their growing use with children supports children’s right to be heard in decisions affecting their care, yet it also raises ethical challenges that general paediatric research ethics guidance does not address in operational detail: sustained cognitive demand, potential emotional distress, assent that can blur under clinical stress, parental influence, clinician–researcher role conflict, and the processing of children’s personal data.

**Methods:**

We developed a prospective ethical governance framework during the preparatory phase of the PREF-ANX Kids study (Preferences for Preoperative Anxiety Management in Children), a planned multicentre DCE examining preferences for nursing interventions to manage preoperative anxiety among children aged 6–12 years undergoing elective surgery in Portuguese public hospitals, their parents, and healthcare professionals. Development combined a pragmatic, non-systematic synthesis of ethical, regulatory, and methodological guidance with iterative structured discussion within an interdisciplinary team. No formal consensus method was applied, and no children or families were involved in development; we identify this as a limitation.

**Framework:**

The framework comprises four domains operating across the research lifecycle: relational participation, cognitive and emotional safeguarding, reflexive professional responsibility, and privacy stewardship. For each domain it specifies planned procedures (continuous assent and dissent monitoring, adapted task burden, structured reflexive documentation, and a prospective data protection impact assessment) together with their normative justification, and it distinguishes elements specific to PREF-ANX Kids from elements potentially adaptable to other paediatric DCEs. Trade-offs between competing ethical and methodological priorities are made explicit rather than resolved silently.

**Conclusions:**

The framework is prospective: it has not yet been implemented or empirically evaluated, and no claims of demonstrated effectiveness are made. It is intended to support transparent ethical planning of DCEs involving children aged 6–12 years and will be assessed during the PREF-ANX Kids study. Future refinement should incorporate co-design with children and families and empirical evaluation of feasibility, acceptability, participant experience, and data quality.

## Background

Children have a recognised right, under Article 12 of the United Nations Convention on the Rights of the Child (UNCRC), to express their views in matters affecting them and to have those views given due weight according to their age and maturity. [1] In healthcare, this right underpins the movement towards person-centred, developmentally responsive care, and towards research that treats children as agents whose preferences carry evidential and moral weight rather than as passive subjects represented solely by adult proxies. [2, 3] Empirical work indicates that children demonstrate greater decisional competence than is often assumed when information is presented in accessible ways. [3, 4] Eliciting children’s preferences systematically therefore serves two connected purposes: it respects developing autonomy, and it generates evidence that can align healthcare services with what children themselves value.

Discrete choice experiments (DCEs) are among the principal quantitative methods for preference elicitation. Developed largely within health economics, a DCE presents participants with a sequence of choice tasks, each requiring a selection between hypothetical alternatives described by attributes (for example, who is present, what a procedure involves, or how long it lasts) whose levels vary systematically; statistical modelling of the observed choices estimates the relative importance participants attach to each attribute. [5, 6] In paediatric perioperative research, a child might choose between scenarios combining parental presence at anaesthesia induction, distraction techniques, waiting-room activities, and preparation approaches. DCEs are attractive in paediatrics because they quantify the trade-offs children are willing to make, rather than merely ranking isolated features. [7, 8].

### Ethical challenges specific to DCEs involving children

The features that make DCEs informative also create ethical challenges when the participants are children, particularly children aged 6–12 years in clinical settings. Six challenges motivated the present framework. First, cognitive burden: repeated hypothetical trade-offs across multiple attributes impose sustained demands that may exceed a child’s developmental or situational capacity, producing fatigue and disengagement. [6, 9] Second, emotional distress: choice scenarios may address anxiety-provoking content (surgery, separation, pain), and participation itself may occur under preoperative stress. [5, 7] Third, the fragility of assent: a child may agree verbally while communicating hesitation non-verbally, and the boundary between autonomous assent and fatigued compliance blurs across successive tasks. [10, 11] Fourth, parental influence: children naturally seek parental reassurance, which can support participation but can also displace the child’s own preferences. [12, 13] Fifth, institutional authority and role conflict: when researchers also hold clinical roles, children and families may perceive participation as connected to care, weakening voluntariness. [14] Sixth, data protection: DCEs process children’s personal and health-related data, whose long-term implications children cannot fully appraise. [15, 16].

General paediatric research ethics guidance (the Declaration of Helsinki, CIOMS Guideline 17, and national frameworks) establishes principles of protection, proportionality, and participation, [3, 17–19] but offers little on how these principles are to be enacted within the specific mechanics of a DCE: the number of choice tasks, dominance tests, piloting, real-time monitoring, or the timing of anonymisation. Published paediatric DCEs, in turn, rarely report such procedures. [9, 20, 21].

### Aim and positioning of this article

This article presents a prospective ethical governance framework developed during the preparatory phase of a single planned study, the PREF-ANX Kids study. It is not a report of empirical findings, and it makes no claim of demonstrated effectiveness: none of the procedures described has yet been implemented or evaluated. Its intended contribution is methodological and normative: to make explicit, before fieldwork begins, the procedures through which established ethical principles will be enacted in one paediatric DCE, the reasoning behind those procedures, and the trade-offs they involve. Throughout, we distinguish elements specific to PREF-ANX Kids, elements potentially adaptable to other paediatric DCEs, and the small number of general principles we consider transferable. We use the term ‘ethical governance framework’ (hereafter ‘the framework’) consistently to refer to this structure.

## Development of the framework

### Study context

The framework was developed for the PREF-ANX Kids study, a planned multicentre cross-sectional DCE examining preferences for nursing interventions to manage preoperative anxiety in children undergoing elective surgery, conducted within a doctoral research programme in nursing. Three participant groups are planned: children aged 6–12 years, their parents or legal guardians, and healthcare professionals involved in perioperative care, recruited across public hospitals in Portugal. The framework addresses primarily the children’s arm, where the challenges enumerated in Sect.  1.1 converge: participation occurs close to surgery, within institutional environments, in the presence of parents, and sometimes facilitated by researchers who also hold clinical roles. Ethical approval for the study protocol has been obtained from the Ethics Committee of ULS Matosinhos (Reference No. 20250099), with applications pending at further participating institutions; no recruitment or data collection has commenced.

### Development process

The framework was developed during the preparatory phase of the study (2025–2026), in parallel with protocol design, ethics committee submissions, and preparation of the Data Protection Impact Assessment (DPIA). Three sources informed its content. First, a pragmatic, non-systematic review of ethical and regulatory guidance: the Declaration of Helsinki (2024 revision), the CIOMS International Ethical Guidelines, the UNCRC, the General Data Protection Regulation (GDPR) and Portuguese Law 58/2019, and literature on paediatric assent and dissent, relational autonomy, vulnerability, and research with children and young people. [1, 3, 10, 12, 15, 18, 19, 22] Second, methodological guidance for DCEs, including ISPOR good research practices, reporting checklists, and published accounts of preference research involving children. [5, 6, 9, 23–25] Third, iterative structured discussion within the research team, whose members contribute expertise in paediatric and perioperative nursing, nursing ethics and clinical supervision, and health economics and DCE methodology, and who are based in Portugal and Sweden.

No formal consensus procedure, such as a Delphi study or nominal group technique, was used. The four domains were derived by grouping the challenges identified in the literature and in the ethics and data-protection submissions into clusters sharing a common object of concern: the child’s participation, the child’s cognitive and emotional state, the researcher’s position, and the child’s data. The clusters were refined across protocol drafts until the team agreed that each planned procedure belonged to exactly one domain. Where priorities conflicted, disagreement was resolved by discussion under an explicit decision rule: the child’s welfare and expressed will take precedence over data completeness (Sect.  4 documents the principal instance). No children, parents, or patient and public involvement contributors participated in development. Given the framework’s emphasis on child-centred safeguards, we regard this as a substantive limitation (Sect.  5.3), and participatory refinement involving children and families is planned.

Overview of the ethical governance framework developed for the PREF-ANX Kids study. The four domains (relational participation, cognitive and emotional safeguarding, reflexive professional responsibility, and privacy stewardship) are interdependent rather than sequential: each is anchored in the same object of concern, the ethical participation of children aged 6–12 years, and each remains active across the five stages of the operational lifecycle shown beneath (design, pilot, recruitment, data collection, dissemination). The lower panel indicates the stage at which each cluster of planned safeguards is first engaged; all four domains require continuous attention throughout.

## The ethical governance framework

**Fig. 1:**
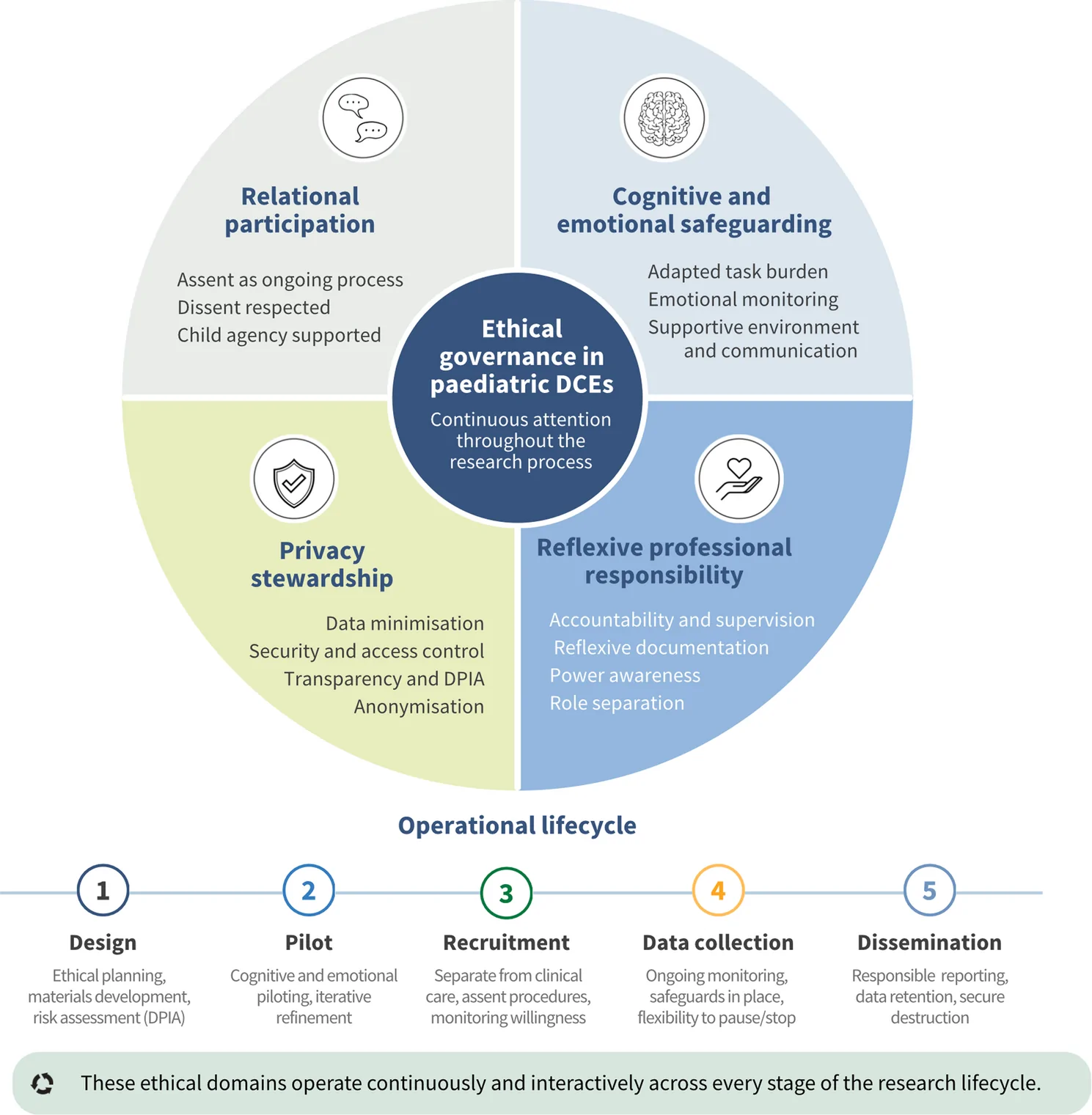
Operational ethical domains in paediatric discrete choice experiments

The framework comprises four domains (relational participation, cognitive and emotional safeguarding, reflexive professional responsibility, and privacy stewardship) that operate continuously and interactively across five lifecycle stages: design, piloting, recruitment, data collection, and dissemination (Fig. 1). Table 1 summarises the challenge each domain addresses and the corresponding planned procedures, and is intended as an orientation device linking challenge, rationale, and response. The subsections below present each domain with its normative justification, distinguishing what is specific to PREF-ANX Kids from what may be adaptable elsewhere.

### Relational participation

Within the framework, assent is treated as an ongoing relational process rather than a single administrative event. [11, 26] In perioperative settings, children may agree to participate while displaying hesitation, fatigue, or reliance on parental reassurance; a signature obtained after parental consent cannot, by itself, establish continued willingness across repeated choice tasks. [10, 27, 28] Researchers will therefore monitor verbal and non-verbal indicators of reduced willingness throughout participation, including repeated reassurance-seeking, disengagement from pictorial tasks, visible withdrawal, and inconsistent responding. [10, 11].

The framework stipulates that a child’s dissent overrides parental permission. This position rests on three converging considerations. Normatively, respect for developing autonomy and Article 12 of the UNCRC require that a child’s expressed will carry weight proportionate to maturity; [1, 3] in research without prospect of direct benefit to the participant, CIOMS guidance, the American Academy of Pediatrics, and the assent literature hold that a child’s deliberate objection should ordinarily be binding, because no therapeutic interest exists to counterbalance it. [11, 18, 29] Relationally, accounts of relational autonomy locate children’s agency within, not against, their family and clinical relationships, requiring researchers to create conditions in which refusal is genuinely available. [12, 13] Practically, dissent expressed under conditions of dependency is often indirect, so procedures must respond to behavioural as well as verbal refusal. [10, 28].

Parental involvement is not treated as inherently problematic. Seeking reassurance, explanation, or emotional support from a parent is developmentally normal for children aged 6–12 years and can enhance understanding and emotional security; supportive involvement of this kind is welcomed, and children may choose to have a parent or trusted caregiver present during participation. The framework distinguishes such support from undue influence: answering on the child’s behalf, pressing continuation despite visible fatigue or distress, or substituting the parent’s preference for the child’s. In the latter situations, researchers will gently redirect attention to the child’s own views and reassess willingness to continue; where hesitancy persists despite parental encouragement, the child’s expressed or observed preference determines whether participation continues.

If a child expresses explicit or implicit dissent mid-protocol, the session will be terminated. Any preference responses collected up to that point will be excluded from analysis and securely destroyed rather than retained; the justification for, and costs of, this decision are examined in Sect.  4. Recruitment and assent discussions will be separated from clinical decision-making whenever possible, children and parents will be told explicitly that refusal or withdrawal will not affect present or future care, and consent for study participation and consent for personal data processing will be documented as separate decisions (Sect.  3.4). [15, 22] Assent discussions will explain, in age-adjusted terms, what participation involves: the nature and number of choice tasks, the expected duration, and the option to pause or stop at any point. [3, 4] Such adaptation extends beyond task materials: explanations of voluntariness, privacy, data use, and the right to stop will differ between children aged approximately 6–8 and 9–12 years, whose understanding of authority, confidentiality, and future data use varies substantially.

### Cognitive and emotional safeguarding

Repeated hypothetical trade-offs impose cognitive demands that preoperative anxiety, unfamiliar environments, and sensory load can amplify. [5–7, 30] Protecting children aged 6–12 years from excessive burden is simultaneously an ethical obligation and a methodological one, because comprehension and attentiveness condition the validity of the preference data produced. [6, 9].

Children will complete fewer choice tasks than adult participants, with a preliminary maximum of eight. This figure was selected pragmatically: published preference studies involving children and young people have typically used conservative task numbers, and methodological guidance recommends restraint where cognitive capacity or situational stress may limit engagement. [6, 8, 9] Direct evidence on the optimal number of tasks for children in perioperative settings is lacking; eight is therefore a starting value to be tested during piloting and reduced if indicated. Each task will include an explicit opt-out alternative so that no child is required to endorse an option they find unacceptable; pilot findings will inform the final design.

Because children aged 6–12 years span wide cognitive and linguistic variation, study materials will be adapted rather than uniform: simplified attribute descriptions, pictorial supports, colour coding, and age-appropriate illustrations, with additional verbal scaffolding anticipated for younger children (approximately 6–8 years) and more autonomous completion for older children (approximately 9–12 years). These materials have not yet been tested. Cognitive pre-testing and think-aloud interviews with children across the age range will assess comprehension during piloting, and materials will be revised iteratively before implementation. [5, 6] Blocking and partial-profile designs will distribute attribute complexity across tasks.

Embedded dominance tests (choice tasks containing one alternative that is logically superior on every attribute) are conventionally used as data-quality checks. [6] The framework additionally assigns them a safeguarding function: failure may indicate misunderstanding, inattention, or fatigue, so a failed dominance test will be interpreted alongside the child’s behaviour, age, and progress through the session, and will prompt the researcher to pause, reassess the child’s comfort and comprehension, and offer clarification, a break, or discontinuation. A failed test is treated as a signal for responsiveness towards the child, not as grounds for excluding the child’s data on quality grounds alone.

Monitoring will be performed by the researcher conducting the session, trained under a written procedure common to all sites in child-centred communication and in a defined set of behavioural indicators: prolonged response latency, repeated requests for clarification, inconsistent use of visual supports, visible frustration or withdrawal, and declining attentiveness. Any of these indicators triggers a structured response sequence (pause, comfort check, clarification, optional break), with discontinuation offered whenever indicators persist. Observations and actions taken will be documented on a standard session record. Consistency across sites will be supported by joint training, calibration exercises during piloting, and periodic review of session records within supervision (Sect.  3.3). No validated quantitative thresholds exist for these indicators; decisions will rest on converging observations, which introduces judgement and potential inter-observer variability (Sect.  5.3).

Throughout, clinical care takes priority over research participation. This ordering follows from the fiduciary character of the clinical relationship and the principle of non-maleficence: the child is owed care as a patient independently of any research role, whereas research participation is discretionary and, in this study, offers no prospect of direct benefit. [18, 31] Where clinical needs and research activities compete (for example, when preparation for anaesthesia coincides with a scheduled session), the research activity will yield, be rescheduled, or be abandoned.

### Reflexive professional responsibility

In PREF-ANX Kids, as in much doctoral and clinically embedded research, data collection will sometimes be undertaken by researchers who hold clinical roles in paediatric care. Dual positioning can support sensitive, trust-based interaction, but it also creates authority dynamics in which children and families may experience participation as connected to their care or to relational loyalty. [14] Where feasible, recruitment and assent discussions will be conducted by researchers not involved in the child’s clinical care; the framework acknowledges that in smaller-scale research such separation will not always be operationally possible.

Where separation is incomplete, reflexivity becomes the principal safeguard. [32, 33] Reflexive documentation after a session cannot, however, neutralise authority perceived within it; during piloting the team will therefore consider review of recruitment and assent interactions by a researcher without direct clinical responsibility for the child, where feasible, to identify wording, timing, or manner that may invite compliance and to refine training before wider recruitment. After each data-collection session, the researcher will complete a brief reflexive memo structured around defined prompts (Box 1), recording perceived authority effects, reassurance-seeking directed at the researcher, parental influence, and any difficulty separating clinical and research roles.

**Table:** 

Box 1. Examples of structured reflexive prompts for paediatric DCE researchers	
• How may my professional role or communication style have influenced the child’s willingness to participate? • Did the child appear to seek reassurance or approval from me during participation? • Were there moments where parental influence appeared to shape the child’s responses or willingness to continue? • Did I interpret any behavioural signs of fatigue, hesitation, or disengagement during participation? • Were there situations in which my responsibilities as a clinician and researcher became difficult to separate? • How might the clinical environment itself have influenced participation dynamics during this interaction?	

Memos and session records will be reviewed at scheduled supervision meetings involving the doctoral researcher and the supervisory team, with site collaborators consulted where site-specific issues arise. A recurring pattern across sessions (for example, repeated reassurance-seeking directed at a clinician-researcher) will trigger review of the affected procedure and, where a change concerns approved protocol elements, an amendment to the relevant ethics committee. Reflexivity is thereby given an explicit pathway from observation to procedural change, rather than remaining a private exercise in self-awareness. [34].

### Privacy stewardship

Privacy stewardship differs from the preceding domains in that its primary mechanisms are institutional rather than interactional. It is retained within the framework for two reasons. First, data protection is continuous with the child’s experience of participation: what children and parents are told about data, namely that consent for participation and consent for data processing are separate decisions and that withdrawal of identifiable data becomes impossible after irreversible anonymisation, forms part of assent and consent communication, and honesty about these limits is a condition of trustworthy participation. [15, 22] Second, children cannot fully appraise the long-term implications of data processing, so protecting their informational future is an obligation owed to their developing autonomy rather than a regulatory requirement alone. [16, 35].

The planned procedures follow the GDPR and Portuguese Law 58/2019. [15, 22] Data will be pseudonymised at the point of collection, with identifiers stored separately under role-based access control and encryption. A DPIA was completed prospectively, in accordance with GDPR Article 35, and shaped design decisions before recruitment: individual-level pseudonymised data will be processed only within Portugal, with Swedish collaborators receiving solely anonymised, aggregated analytical outputs, thereby removing cross-border transfer of personal data. Data will remain pseudonymised during collection and verification, preserving the possibility of withdrawal and correction, and will be irreversibly anonymised only after final analytical datasets are prepared, after which selective withdrawal is no longer technically feasible; this sequence and its consequences will be explained during consent and assent discussions. Retention periods are predefined, with progressive anonymisation and secure destruction in accordance with institutional policy.

Table 1 consolidates these challenges and planned responses; readers applying the framework elsewhere should treat the responses as design options requiring local justification and piloting rather than as prescriptions.

**Table 1:** Principal ethical challenges and governance responses in paediatric DCEs

Ethical challenge	Why it matters in paediatric DCEs	Operational response under the proposed framework
Repeated hypothetical choice tasks	May increase fatigue, reduced concentration, and disengagement during participation	Reduced number of choice tasks, optional pauses, cognitive piloting
Preoperative anxiety	May affect emotional comfort, comprehension, and willingness to participate	Emotional monitoring, supportive communication, flexible participation procedures
Institutional authority and clinical dependency	Children and families may feel reluctant to refuse participation	Separation between recruitment and clinical care interactions, emphasis on voluntariness
Abstract trade-offs and complex attributes	Children may experience difficulty understanding hypothetical scenarios	Simplified language, pictorial supports, colour coding, think-aloud piloting
Parental influence during participation	Children’s responses may become shaped by parental reassurance or expectations	Ongoing assent monitoring, attention to verbal and non-verbal dissent
Emotional distress during participation	Anxiety-related discussions may trigger withdrawal or discomfort	Continuous monitoring, option to discontinue participation, supportive researcher presence
Processing of sensitive personal data	Children may not fully understand long-term implications of data use	GDPR-compliant governance, pseudonymisation, restricted access procedures, Data Protection Impact Assessment (DPIA)
Dual clinician-researcher roles	Professional authority may unintentionally influence participation decisions	Reflexive documentation, separation of roles whenever feasible, supervisory oversight
Dominance test failure or inattentive responding	May indicate excessive burden or compromised participation quality	Pause/ reassessment procedures, clarification, optional discontinuation

## Ethical tensions and trade-offs

The safeguards described above involve genuine trade-offs between ethical and methodological priorities, which the framework makes explicit rather than resolving silently.

### Burden reduction against task fidelity

Restricting children to a small number of simplified, pictorially supported tasks reduces cognitive and emotional load, but it also constrains the number of attributes and levels that can be estimated, reduces statistical efficiency, and risks altering the meaning of attributes through simplification. The framework accepts a narrower and less efficient design as the price of proportionate participation, and relies on piloting to identify the least distorting simplifications.

### Responsiveness to dissent against data completeness

Terminating sessions on behavioural indicators, and destroying partial data after dissent, protect children but reduce sample completeness and remove information that could otherwise support attrition analysis. Destruction was chosen over retention of partial data on the reasoning that a child who has withdrawn assent has withdrawn it for the data already given; retaining data collected up to the moment of dissent would subordinate the child’s expressed will to analytic convenience. [11, 29] To preserve transparency and to detect design-related sources of burden during piloting, the standard session record will note only minimal coded safety metadata: the session stage, the task number, and, where observable, the broad reason for discontinuation, without the child’s choices, preference responses, or free-text content. These metadata will not contribute to preference modelling, and the number and timing of discontinuations will be reported.

### Parental presence against parental influence

Allowing a parent to be present supports emotional security but increases opportunities for influence. The framework accepts this trade-off in favour of the child’s comfort, managing rather than eliminating influence through the redirection procedures described in Sect.  3.1.

### Clinician involvement against role conflict

Clinically experienced researchers may detect distress earlier and respond more skilfully, yet their involvement intensifies authority dynamics. Where separation of roles is infeasible, the framework substitutes reflexive documentation and supervisory review, a weaker safeguard adopted for feasibility and identified as such.

### Proportionality across contexts

Table 2 distinguishes safeguards we regard as essential in any paediatric DCE from enhanced safeguards feasible mainly within larger or specialised research infrastructures. The distinction is driven primarily by participant vulnerability rather than by resources alone: younger children, heightened perioperative anxiety, or cognitively demanding designs raise the level of safeguarding required. A context unable to provide safeguards proportionate to the vulnerability of its participants should modify the design or refrain from proceeding: research is optional; the safety of participants is not.^18^,^36^.

**Table 2:** Examples of proportionate ethical safeguards in paediatric DCEs

Essential safeguards	Enhanced safeguards
Ongoing assent monitoring	Independent assent facilitators
Visual distress monitoring	Dedicated reflexive supervision
Flexible pause/ discontinuation procedures	Separate recruitment personnel
Simplified visual supports	Extended cognitive piloting
Separate consent for data processing	Multi-layer institutional oversight

## Discussion

### What the framework adds

The framework does not propose new ethical principles. Continuous assent, burden minimisation, voluntariness, and data protection are established requirements of paediatric research ethics. [3, 11, 18, 19] Its intended contribution lies elsewhere, in three respects. First, integration: the four domains assemble safeguards usually treated separately (assent practice, cognitive testing, reflexivity, and data governance) into a single structure spanning the research lifecycle. Second, DCE specificity: the framework attaches ethical functions to method-specific features, such as dominance tests reinterpreted as safeguarding triggers, task-number decisions treated as ethical rather than purely statistical decisions, and anonymisation timing weighed against withdrawal rights. Third, prospective transparency: publishing the procedures and their justification before fieldwork exposes them to critique while revision remains possible and creates a benchmark against which implementation can later be compared. [9, 20].

### Scope and transferability

Three tiers should be distinguished. Specific to PREF-ANX Kids are the operational parameters: the eight-task limit, the Portuguese data-localisation arrangement, the particular indicator set, and the supervisory structure of a doctoral programme. Potentially adaptable to other paediatric DCEs, subject to local piloting and regulatory adjustment, are the procedural mechanisms: continuous assent monitoring with a structured response sequence, dominance tests as safeguarding triggers, staged anonymisation, and structured reflexive documentation. General, in our view, are only the underlying principles: that dissent by a child in research without prospect of direct benefit should be binding, that clinical care takes priority over research, and that safeguarding must be proportionate to vulnerability; these are grounded in existing guidance rather than original to this framework. [1, 11, 18, 29] Claims beyond this third tier would exceed the available evidence: the framework’s transferability, like its feasibility, remains to be demonstrated.

### Limitations

The principal limitation is the framework’s prospective status: no implementation, feasibility, acceptability, or outcome data exist, and the language of this article is therefore intentional (‘will’, ‘is designed to’) rather than evaluative. Second, no children, parents, or patient and public involvement contributors participated in development. A framework designed exclusively by adults may embed adult assumptions about what children aged 6–12 years experience as acceptable burden, distress, or meaningful support, a tension with the participatory principle it invokes; participatory refinement (co-design of study materials with children and families, and qualitative evaluation of their experiences of the procedures) is planned within the PREF-ANX Kids programme. Third, several operational decisions, including the eight-task limit, the behavioural indicator set without validated thresholds, and the essential-enhanced classification in Table 2, rest on limited evidence and pragmatic judgement, and real-time interpretation of behavioural indicators may vary between observers in multicentre implementation despite joint training and calibration. Fourth, the framework is context-bound: a perioperative setting, children aged 6–12 years, Portuguese public hospitals, and the European data protection regime; adaptation would be required in settings with different legal frameworks, institutional infrastructures, cultural expectations, or resource constraints. Finally, no set of procedures removes the power asymmetries inherent in paediatric healthcare research; the framework aims to make them visible and manageable, not to abolish them.

The essential-enhanced distinction in Table 2 is intended as a vulnerability-driven planning aid, not a hierarchy of ethical importance; its categories were assigned by team judgement and will be revisited in the light of implementation experience.

## Conclusions

This article has presented a prospective ethical governance framework developed for the PREF-ANX Kids study, organised around four domains (relational participation, cognitive and emotional safeguarding, reflexive professional responsibility, and privacy stewardship) and around the trade-offs that connect them. The framework has not been implemented or evaluated. Whether it improves children’s experience of participation, supports ethical conduct, proves feasible within clinical workflows, and sustains data quality are empirical questions that the PREF-ANX Kids study is designed to begin answering.

Its present value is deliberately modest: to make explicit, and open to criticism, the reasoning by which one research team intends to translate established ethical principles into the specific mechanics of a DCE involving children aged 6–12 years. Future work will incorporate children’s and families’ perspectives through co-design and will evaluate the framework empirically; only on that basis could stronger claims about its utility, or its transferability to other paediatric preference research, be justified.

## Data Availability

No datasets were generated or analysed for the present manuscript. Data arising from the future PREF-ANX Kids study will be managed in accordance with approved ethical and data-protection procedures. Given that the study will involve children aged 6-12 years in a clinical context, individual-level data will not be made publicly available unless they can be fully anonymised in accordance with applicable ethical, legal, and institutional requirements. Aggregated or anonymised materials may be made available from the corresponding author upon reasonable request, where legally and ethically permissible and subject to relevant approvals.

## References

[1] United Nations. Convention on the Rights of the Child. New York: United Nations; 1989.

[2] Damsma Bakker A, van Leeuwen R, Roodbol P. Ethical considerations regarding the inclusion of children in nursing research. Nurs Ethics. 2021;28(1):106–17. 10.1177/0969733020948120 . Epub 2020 Sep 30. PMID: 32996358. 32996358

[3] Alderson P, Morrow V. The Ethics of Research with Children and Young People: A Practical Handbook. London: SAGE; 2011. 10.4135/9781446268377.

[4] Grootens-Wiegers P, Hein IM, van den Broek JM, de Vries MC. Medical decision-making in children and adolescents: developmental and neuroscientific aspects. BMC Pediatr. 2017;17(1):120. 10.1186/s12887-017-0869-x . PMID: 28482854; PMCID: PMC5422908. 28482854 PMC5422908

[5] Bridges JF, Hauber AB, Marshall D, Lloyd A, Prosser LA, Regier DA, Johnson FR, Mauskopf J. Conjoint analysis applications in health – a checklist: a report of the ISPOR Good Research Practices for Conjoint Analysis Task Force. Value Health. 2011;14(4):403–13. Epub 2011 Apr 22. PMID: 21669364. 21669364 10.1016/j.jval.2010.11.013

[6] Reed Johnson F, Lancsar E, Marshall D, Kilambi V, Mühlbacher A, Regier DA, Bresnahan BW, Kanninen B, Bridges JF. Constructing experimental designs for discrete-choice experiments: report of the ISPOR Conjoint Analysis Experimental Design Good Research Practices Task Force. Value Health. 2013 Jan-Feb;16(1):3–13. 10.1016/j.jval.2012.08.2223. PMID: 23337210. 23337210

[7] Soekhai V, de Bekker-Grob EW, Ellis AR, Vass CM. Discrete choice experiments in health economics: past, present and future. Pharmacoeconomics. 2019;37(2):201–226. 10.1007/s40273-018-0734-2. PMID: 30392040. PMC6386055 30392040

[8] Williams G, Lorgelly P. The application of discrete choice experiments eliciting young peoples’ preferences for healthcare: a systematic literature review. Eur J Health Econ. 2022;23(4):603–19. 10.1007/s10198-021-01389-8. PMC10290600 36169764

[9] Campoamor N, Clarke PM, Loo CK, Cohen-Almagor R, Kelleher ST, Mooney SJ, Bridges JF. Pretesting Discrete-Choice Experiments: A Guide for Researchers. Patient. 2024;17(5):491–502. 10.1007/s40271-024-00695-0. PMID: 38769309. PMC10894089 38363501

[10] Spriggs M. Children and bioethics: clarifying consent and assent in medical and research settings. Br Med Bull. 2023;145(1):110–9. 10.1093/bmb/ldac038 . PMID: 36723953; PMCID: PMC10075240. 36723953 PMC10075240

[11] Katz AL, Webb SA, AAP Committee on Bioethics. Informed Consent in Decision-Making in Pediatric Practice. Pediatrics. 2016;138(2):e20161485. 10.1542/peds.2016-1485. PMID: 27456510. 27456510

[12] Mackenzie C, Stoljar N, editors. Relational Autonomy: Feminist Perspectives on Autonomy, Agency, and the Social Self. New York: Oxford University Press; 2000.

[13] Matthiesen A, Larsen BH, Rosen MH, Søndergaard J, Frederiksen K, Nielsen CP. Relational autonomy in pediatric healthcare: A scoping review. Nurs Ethics. 2025;32(1):21–39. doi: 10.1177/09697330241230786. PMID: 38362699. 10.1177/09697330251366601 PMC12907465 40940033

[14] Geddis-Regan AR, Thomson RG, Watson MS, Exley C. Navigating the Dual Role of Clinician-Researcher in Qualitative Dental Research. JDR Clin Trans Res. 2021;6(1):4–11. doi: 10.1177/2380084420966618. PMID: 33078608. 10.1177/2380084421998613 33618559

[15] Regulation (EU). 2016/679 of the European Parliament and of the Council of 27 April 2016 on the protection of natural persons with regard to the processing of personal data (General Data Protection Regulation). Official Journal of the European Union L119/1. Available from: https://eur-lex.europa.eu/legal-content/EN/TXT/?uri=CELEX%3A32016R0679

[16] Livingstone S, Third A. Children and young people’s rights in the digital age: an emerging agenda. New Media Soc. 2017;19(5):657–70. 10.1177/1461444816686318.

[17] Bagnasco A, Cadorin L, Barisone M, Bressan V, Iemmi M, Prandi M, Timmins F, Watson R, Sasso L. Ethical dimensions of paediatric nursing: A rapid evidence assessment. Nurs Ethics. 2018;25(1):111–22. Epub 2016 Mar 22. PMID: 27005952. 27005952 10.1177/0969733016631161

[18] Council for International Organizations of Medical Sciences (CIOMS). International Ethical Guidelines for Health-Related Research Involving Humans. 4th ed. Geneva: CIOMS; 2016. 40523065

[19] World Medical Association. Declaration of Helsinki: Ethical Principles for Medical Research Involving Human Participants. Adopted by the 75th WMA General Assembly, Helsinki, Finland, October 2024. JAMA. 2025;333(1):71–4. 10.1001/jama.2024.21972. 39425955

[20] Illes J, Lawson A, McDonald PJ. Ethical Considerations for Discrete Choice Experiments with Caregivers. J Empir Res Hum Res Ethics. 2022;17(4):426–430. doi: 10.1177/15562646221112339. Epub 2022 Jul 18. PMID: 35849082; PMCID: PMC9398987. 10.1177/15562646221112339 PMC9398987 35849082

[21] Michaels-Igbokwe C, Yamamoto C, Marshall DA. Methods for Conducting Stated Preference Research with Children and Adolescents in Health: A Scoping Review of the Application of Discrete Choice Experiments. Patient. 2021;14(5):539–52. 10.1007/s40271-021-00518-6. 34008164

[22] Lei n.º 58/2019 de 8 de agosto. Assegura a execução, na ordem jurídica nacional, do Regulamento (UE) 2016/679 do Parlamento Europeu e do Conselho, de 27 de abril de 2016, relativo à proteção das pessoas singulares no que diz respeito ao tratamento de dados pessoais e à livre circulação desses dados. Diário da República 1.ª série, N.º 151:3–40.

[23] International Committee of Medical Journal Editors. Recommendations for the Conduct, Reporting, Editing, and Publication of Scholarly Work in Medical Journals. Updated 2023. Available from: https://www.icmje.org/recommendations/10.12771/emj.2024.e48 PMC12093628 40704003

[24] Ride J, Norman R, Viney R, Lancsar E, Donaldson C, Fiebig DG. A Reporting Checklist for Discrete Choice Experiments in Health: The DIRECT Checklist. Pharmacoeconomics. 2024;42(3):265–278. 10.1007/s40273-023-01338-w. PMID: 38097865. PMC11405421 39227559

[25] Pestana-Santos M, Neves H, Esteves IM, Rodrigues MA, Rocha DM. Nonpharmacological interventions used in the perioperative period to prevent anxiety in adolescents: a scoping review. JBI Evid Synth. 2021;19(12):3348–3399. 10.11124/JBIES-20-00452. PMID: 34016845. 34038923

[26] Hester DM, Miner SA. Consent and Assent in Pediatric Research. Pediatr Clin North Am. 2024;71(1):83–92. 10.1016/j.pcl.2023.08.003. Epub 2023 Sep 11. PMID: 37973309. 37973309

[27] Cayouette F, Beauchamp MH, Labrecque M, Bhatt M, Trottier ED, Faraj S, Roy M, Gravel J. Operationalization of assent for research participation in pre-adolescent children: a scoping review. BMC Med Ethics. 2022;23(1):20. 10.1186/s12910-022-00760-7. PMID: 35232423. PMC9632024 36329421

[28] Van Goidsenhoven L, Mortelmans D, De Graef A. Relational ethics, informed consent, and informed assent in participatory research with children with complex communication needs. Dev Med Child Neurol. 2022;64(10):1184–1190. 10.1111/dmcn.15307. PMID: 35143069. 35665498

[29] Wendler D, Shah S. Should children decide whether they are enrolled in nonbeneficial research? Am J Bioeth. 2003;3(4):1–7. 10.1162/152651603322614382. PMID: 14744301. 14744301

[30] Rogers HJ, Mulhern B, Gillard S, Rowen D. Discrete choice experiments or best-worst scaling? A qualitative study to determine the suitability of preference elicitation tasks in research with children and young people. J Patient Rep Outcomes. 2021;5(1):41. 10.1186/s41687-021-00309-3. PMID: 33956281. PMC7947050 33689059

[31] Beauchamp TL, Childress JF. Principles of Biomedical Ethics. 8th ed. New York: Oxford University Press; 2019.

[32] Montreuil M, Gohier B, Racine E. A Review of Approaches, Strategies and Ethical Considerations in Participatory Research With Children. Int J Qual Methods. 2021;20:1–12. 10.1177/16094069211004397.

[33] Finlay L. Outing the researcher: the provenance, process, and practice of reflexivity. Qual Health Res. 2002;12(4):531–45. 10.1177/104973202129120052. 11939252

[34] Tschudin V. Ethics in Nursing: The Caring Relationship. 3rd ed. Edinburgh: Butterworth-Heinemann; 2003.

[35] Facca D, Smith MJ, Shelley J, Lizotte D, Donelle L. Exploring the ethical issues in research using digital data collection strategies with minors: A scoping review. PLoS One. 2020;15(6):e0237875. 10.1371/journal.pone.0237875. PMID: 32497065. PMC7451523 32853218

[36] Victor E, Bhatt DL. Vulnerability in practice: Peeling back the layers, avoiding triggers, and preventing cascading effects. Bioethics. 2022;36(7):510–518. 10.1111/bioe.13051. PMID: 35426163. PMC9886167 35481605

